# Ligand-based CAR-T cell: Different strategies to drive T cells in future new treatments

**DOI:** 10.3389/fimmu.2022.932559

**Published:** 2022-09-12

**Authors:** Alejandro Ramírez-Chacón, Sergi Betriu-Méndez, Ariadna Bartoló-Ibars, Azucena González, Mercè Martí, Manel Juan

**Affiliations:** ^1^ Immunology Unit, Department of Cellular Biology, Physiology and Immunology, Universitat Autònoma de Barcelona (UAB), Cerdanyola del Vallès, Spain; ^2^ Laboratory of Cellular Immunology, Institute of Biotechnology and Biomedicine (IBB), Cerdanyola del Vallès, Spain; ^3^ Immunology Department, Hospital Clínic de Barcelona, Centre de Diagnòstic Biomèdic (CDB), Barcelona, Spain; ^4^ Immunology Department, Institut d'Investigacions Biomèdiques August Pi i Sunyer (IDIBAPS) – Fundació Clínic per a la Recerca Biomèdica (FCRB) Universitat de Barcelona (UB), Barcelona, Spain; ^5^ Immunology Department, Hospital Sant Joan de Déu, Barcelona, Spain

**Keywords:** T cells, chimeric antigen receptor (CAR), ligands, receptor, antigen, CAAR, BAR

## Abstract

Chimeric antigen receptor (CAR)-based therapies are presented as innovative treatments for multiple malignancies. Despite their clinical success, there is scientific evidence of the limitations of these therapies mainly due to immunogenicity issues, toxicities associated with the infusion of the product, and relapses of the tumor. As a result, novel approaches are appearing aiming to solve and/or mitigate the harmful effects of CAR-T therapies. These include strategies based on the use of ligands as binding moieties or ligand-based CAR-T cells. Several proposals are currently under development, with some undergoing clinical trials to assess their potential benefits. In addition to these, therapies such as chimeric autoantibody receptor (CAAR), B-cell receptor antigen for reverse targeting (BAR), and even chimeric human leukocyte antigen (HLA) antibody receptor (CHAR) have emerged, benefiting from the advantages of antigenic ligands as antibody-binding motifs. This review focuses on the potential role that ligands can play in current and future antitumor treatments and in other types of diseases, such as autoimmune diseases or problems associated with transplantation.

## Introduction

Immunotherapy using autologous genetically engineered chimeric antigen receptor (CAR) T (CAR-T) cells is widely emerging as one of the major breakthroughs for treating cancer. The aim of these therapies is focused on driving T-cell cytotoxicity specifically against tumor antigens in cancer cells (1, 2). However, the identification of suitable targeted tumor-associated antigens (TAAs) remains a challenge nowadays due to life-threatening toxicity derived from off-tumor on-target antigen recognition (3–5).

Various approaches are being proposed for both hematological and non-hematological malignancies (6). Remarkable antitumor responses have been achieved from anti-CD19 CAR-T therapies against B-cell acute lymphoblastic leukemia (B-ALL) and other refractory B-cell malignancies, demonstrated in pivotal clinical trials (7–11). This has led to the approval by Food and Drug Administration (FDA) and/or European Medicines Agency (EMA) of several CD19-directed products, such as tisagenlecleucel (KYMRIAH^®^, Novartis) (12), axicabtagene ciloleucel (YESCARTA^®^, Kite Pharma-Gilead) (13), and lisocabtagene maraleucel (BREYANZI^®^, Juno Therapeutics-Celgene-BMS) (14) for treating large B-cell lymphoma and brexucabtagene autoleucel (TECARTUS^®^, Kite Pharma-Gilead) (15) for treating relapsed/refractory mantle cell lymphoma. In this context, our group obtained the first European-developed CAR-T approved by the Spanish Agency of Medicine [*Agencia Española del Medicamento y Productos Sanitarios* (AEMPS)] with the authorization of ARI-0001 (at *Hospital Clínic de Barcelona*) administration for relapsed or refractory CD19+ B-ALL in adult patients (16). Another CAR-T treatment recently approved by the FDA is idecabtagene vicleucel (ABECMA^®^, Celgene-BMS) based on the recognition of B-cell maturation agents (BCMAs) for treating multiple myeloma (MM) (17). Besides approved therapies, ciltacabtagene autoleucel (of CARTITUDE clinical trials, Janssen-Johnson&Johnson) (18) is also a BCMA-directed CAR-T product expected to be authorized for MM treatment.

Despite this range of validated products, researchers are striving to broaden the clinical benefit of CAR-T cells while exploring new cutting-edge applications. Nevertheless, resistance mechanisms, such as T-cell exhaustion, immunosuppression, or antigen loss, carrying to relapse and therapy failure, have been reported during these trials (19, 20). In fact, an estimated 30% of relapses after anti-CD19 therapy were related to antigen loss (20). This highlights the need to improve or fine-tune CAR-T therapies to avoid tumor escape by identifying novel tumor antigen targets, testing various CAR-expressing cells as CAR-Natural killer (NK), and, most importantly, enhancing the CAR molecule itself.

**Figure 1:**
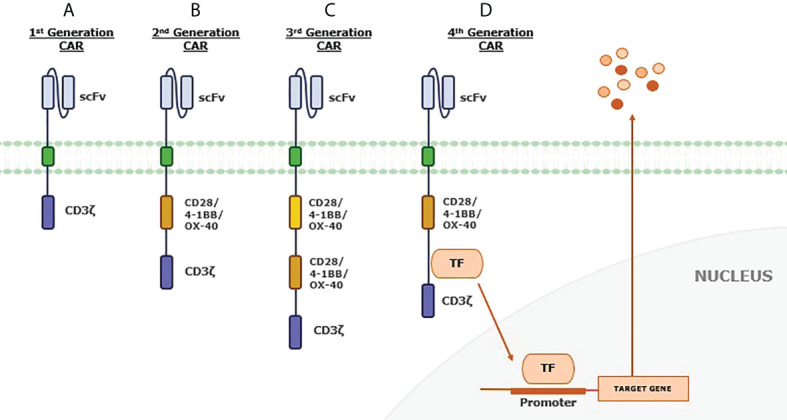
Chimeric antigen receptor (CAR) generations. **(A)** First-generation CAR includes a single-chain variable fragment (scFv) extracellular region and a T-cell activation domain. This minimal structure can recognize the antigen in an HLA-independent manner. By adding a costimulatory domain, **(B)** second-generation CAR is more able to expand and persist due to this second signal. **(C)** The third-generation CAR has an additional costimulatory signaling domain to increase proliferation, survival, and activity of engrafted T cells. Recently, **(D)** the fourth-generation CAR has been developed to include extra genes, such as recognition domains for transcription factors involved in mediating signal transduction. The idea is to modulate the effect of the CAR, facing an immunosuppressive tumor microenvironment by cytokine production or other additional effects.

The minimal structure of a CAR consists of an extracellular antigen recognition domain, tethered to a hinge domain followed by a transmembrane region, and an intracellular signaling domain (CD3ζ). Additionally, different generations can be found according to the number of modular intracellular costimulatory domains (mainly 4-1BB, CD28, or OX-40) (1, 21) **(** **Figure 1** **)**.

Autologous T cells expressing CAR molecules are activated upon non-HLA-restricted ligand recognition, subsequent posttranslational phosphorylation of CD3ζ is produced, and the zeta-chain-associated protein kinase 70 (Zap-70) is recruited, inducing the assembly of downstream proteins. Meanwhile, CAR-costimulatory regions can activate Phosphoinositide 3-kinase (PI3K)/Protein kinase B (AKT), tumor necrosis factor (TNF) receptor-associated factor 2 (TRAF2)/Mitogen-activated protein kinase (p38MAPK), and c-Jun N-terminal kinase (JNK) pathways. Finally, CAR functional domains enable the CAR to integrate all downstream signaling pathways that end up with the expression and activation of transcriptional modulators—Nuclear factor kappa B (NF-κB), Nuclear factor of activated T-cells (NFAT), Signal transducer and activator of transcription 3 (STAT3), Activator protein 1 (AP-1)—to drive the effector function of CAR-T cells (22, 23).

**Figure 2:**
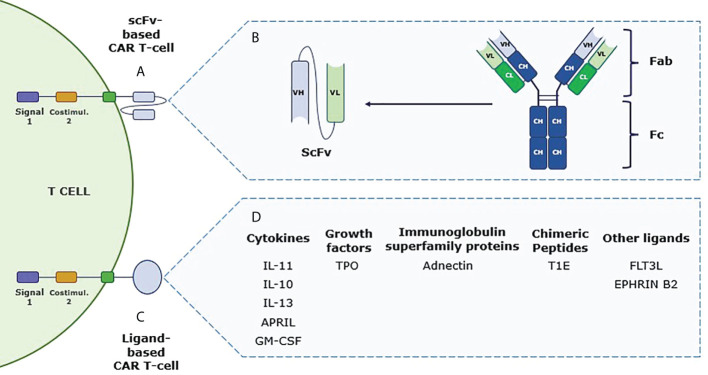
Schematic differences between conventional chimeric antigen receptor T (CAR-T) cell and ligand-based CAR-T cell. **(A)** Second-generation conventional CAR-T cell structure, including T-cell activation domain (signal 1) and costimulatory regions. The recognition domain of this CAR-T is composed of the single-chain variable fragment (scFv) that allows direct interaction with the tumor-associated antigen to trigger the antitumor response. **(B)** Schematic representation of the different chains that form an antibody and how the scFv domain is obtained from the variable heavy and light chains of a monoclonal antibody to be used in the CAR-T structure. **(C)** Structure of a second-generation ligand-based CAR-T cell that shares the same domains as mentioned with scFv-based CAR-T cells but incorporating a ligand as a target recognition domain. **(D)** Immune cytokines, growth factors, immunoglobulin superfamily proteins, and chimeric peptides, among others, are listed as potential molecules to be used as ligand-based CAR-T cell recognition domains (see some abbreviations in **Table 1**).

Largely, the extracellular antigen recognition domain is a single-chain variable fragment (scFv) composed of a variable heavy chain (V_H_) and a variable light chain (V_L_), which are usually derived from a murine monoclonal antibody (moAb), joined by a linker region (1) **(** **Figure 2** **)**. Due to the scFv murine nature, immunogenicity is one of the major issues regarding *in vivo* long-term expression of CAR-T cells (24, 25). Moreover, aggregation and instability can lead to poor persistence and loss of effectiveness (26). Even though the moAb characteristics of scFv allow potential benefits for the CAR-T therapy, cell persistence, antitumor response efficiency, and off-tumor on-target toxicity need to be improved, and thus, innovative approaches can bring improvements in this regard.

## Changing the extracellular target-binding region, changing the “CAR” concept to other but similar receptors

Optimized design of every region of a synthetic CAR has been shown to be relevant to its clinical success (27). Different strategies are being developed to increase CAR-T cell response, one of which is proposing a new extracellular target-binding region (28). Given this, non-antibody-based strategies are being proposed as a promising improvement for CAR-T cell therapies based on the interaction of surface receptors with their natural ligands (29). Specifically, ligand-based CAR-T cells take benefit from the receptor-binding domain of soluble molecules, i.e., cytokines, growth factors, immunoglobulin superfamily proteins, or chimeric peptides for targeting TAA to induce the antitumor response (26) **(** **Figure 2** **)**. Similar to scFv-based CAR-T cells, the ligand recognition is in an HLA-independent manner. Additionally, the downstream signaling T-cell activation is maintained in the ligand-based CAR-Ts as all four generations can be found, but most approaches use the second- and third-generation CARs **(** **Table 1** **)**.

**Table 1:** List of preclinical studies and current clinical trials using ligand-based CAR-T cells in course.

	References	Ligand	Target	CAR structure	Disease
*Preclinical studies*	(30)	IL-11	IL11-Rα	IL11 - CD28 -CD3 ζ	OS and lung metastases
	(31)	Adnectin	EGFR	Adnectin- CD28 - 4-1BB - CD3 ζ	Lung cancer
	(32)	FLT3L	FLT3	FLT3L - 4-1BB - CD3 ζ	AML
	(33)	GM-CSF	GMR	GM-CSF - CD28 - CD3 ζ	AML, JMML
	(34)	EPHRIN B2	EPHB4	EPHRIN B2 -CD28 - CD3 ζ	RMS
	(35)	Tri-APRIL	BCMA/TACI	Tri-APRIL - 4-1BB - CD3 ζ	MM
	(36)	TPO	MPL R	TPO – CD28 - CD3 ζ	AML
	(37)	IL-10	IL-10R	IL-10 - 4-1BB - CD3 ζ	AML
	(38)	CD27	CD70	CD27- CD3 ζ	Diffuse large B-cell lymphoma, follicular lymphoma, AML
*Clinical trials*	NCT02208362	IL-13	IL-13Rα2	IL-13(E13Y) - 4-1BB - CD3 ζ	Glioma
	NCT01818323	T1E	ErbB 1-4	T1E - CD28 - CD3 ζ	HNSCC
	NCT03287804	APRIL	BCMA/TACI	APRIL - CD28 - OX40 - CD3 ζ	MM
	NCT04661384	IL-13	IL-13Rα2	IL-13(E13Y) - 4-1BB - CD3 ζ	Leptomeningeal glioblastoma, Ependymoma or medulloblastoma

OS, osteosarcoma; EGFR, epidermal growth factor receptor; FLT3L, FMS-like tyrosine kinase 3 ligand; FLT3, FMS-like tyrosine kinase 3; AML, acute myeloid leukemia; GM-CSF, granulocyte-monocyte colony-stimulating factor; GMR, granulocyte-monocyte colony-stimulating factor receptor; JMML, juvenile myelomonocytic leukemia; EPHB4, ephrin type-B receptor 4; RMS, rhabdomyosarcoma; APRIL, a proliferation-inducing ligand; BCMA, B-cell maturation antigen; TACI, transmembrane activator and calcium-modulator and cyclophilin ligand interactor; TPO, thrombopoietin; MPLR, myeloproliferative leukemia receptor; MM, multiple myeloma; HNSCC, head and neck squamous cell carcinoma. The clinical trials are collected from clinicaltrials.gov.

One of the main constraints lies in finding the right ligand for the CAR structure, as the pool of candidates is still limited. Moreover, its target must be tumor specific or highly expressed while having minimal presence in normal tissues, making it necessary to optimize the proposals as much as possible. Worldwide, a considerable number of preclinical studies have been published and clinical trials are currently ongoing, validating suitable ligands and elucidating their clinical potential **(** **Table 1** **)**.

## Ligand-based Chimeric Antigen Receptor (CAR)-T cell preclinical studies

Over the recent years, efforts have been focused on proving CAR-T success against solid tumors, but several difficulties have arisen such as finding a specific TAA, insufficient cell expansion after recognition, tumor penetration, and evasion mechanisms. These obstacles for CAR-T therapies need to be overcome (39–41). Indeed, different approaches are being evaluated, including ligand-based strategies.

### IL-11-based CAR-T cell

The IL-11/IL-11Rα signaling pathway is involved in several biological activities, and it is supposed to induce an antiapoptotic effect *via* STAT3 activation (42, 43). It has been shown that human IL-11Rα is overexpressed in several types of cancer, including osteosarcoma (OS) and lung-associated metastases. Immunohistochemistry results from Huang et al. (30) showed that four different OS cell lines overexpress IL-11Rα within 20%–60% and 14 of 16 patients were positive for IL-11Rα in their OS lung metastasis samples. In contrast, IL-11Rα was not expressed in the surrounding normal lung tissue or other essential tissues (30). OS treatment has been stagnant during these years, and finding new treatments is still needed (44). Because of its orphan disease condition, IL-11Rα was proposed as a suitable candidate for CAR-T therapy (45).

A second-generation CAR-T was designed using IL-11 peptide (CGRRAGGSC) as the extracellular domain (46). *In vitro*, IL-11Rα-CAR-T cells were cytotoxic to four different OS cell lines compared with control T cells. After *in vitro* injection in OS mouse models, engineered T cells accumulated in lung metastasis nodules that resulted in selective tumor cell lysis and tumor regression, with no visible lung metastases in three of the five mice treated compared with controls (30).

### Adnectin-based CAR-T cell

Adnectin is derived from the 10th type III domain of human fibronectin (10Fn3) (47). The 10Fn3 domain interacts with integrins and belongs to the immunoglobulin superfamily. Its structure is close to the antibody variable domain but with better stability and no dependence on disulfide bonds (48). This feature allows the manipulation of this domain to generate mutants with different interactions. Hence, a similar scFv structure, increased stability, and human nature make adnectin an interesting candidate for ligand-based CAR-T therapies (31).

One of the target membrane surface receptors to direct adnectin-CAR-T cells is the epithelial growth factor receptor (EGFR). This receptor has tyrosine kinase activity that governs fundamental cellular processes, including proliferation, cell migration, metabolism, and survival (49). Moreover, it is one of the most suitable candidates targeted in cancer therapies, since it is overexpressed in several tumors such as breast, lung, and head and neck (50).

Han et al. (31) designed CETUX-CAR (scFv derived from cetuximab) and adnectin-CAR-T cells targeting EGFR (both third-generation CAR-T) to compare their activity (31). Ligand-based CAR-T therapy was developed after revising a previous work by Emanuel et al. (51) to generate adnectin clones for this aim. Four adnectin clones were evaluated (E1, E2, E3, E4) with different binding affinities. E3 was considered the most eligible (51). In comparison to CETUX-CAR-T therapy, E3 CAR-T cell displayed relatively lower binding affinity toward EGFR but higher selectivity against EGFR-overexpressing cancer cells. Nevertheless, it has comparable reactivity, cytotoxicity, and hence antitumor response when incubated with lung carcinoma H292 cells (31). These characteristics expect new broad opportunities to selectively target EGFR-positive tumor cells, avoiding classical issues of classic CAR-T therapies, which will be discussed later.

### EPHB4-based CAR-T cell

Ephrin type-B receptor 4 (EPHB4), a member of the family of receptor tyrosine kinases (RTKs), is ubiquitously expressed in distinct types of malignancies as rhabdomyosarcoma (RMS). EPHB4 expression is negligible in vital tissues except of a weak expression in normal placenta cells (52, 53). Differences in ligand-dependent or ligand-independent activation of EPHB4 have been reported, being stimulation without ligand binding the one that leads to cell growth and transformation. In RMS and other malignancies, EPHRIN B2 interaction with EPHB4 may induce apoptosis and lack of proliferation (54). Based on the fact that EPHRIN B2 is a unique ephrin ligand that interacts with EPHB4, a second-generation CAR-T cell with an extracellular portion of EPHRIN B2 can be considered for RMS treatment (55).

*In vitro* robust and sustained killing activity against RMS, OS, and triple-negative breast cancer (TNBC) cells was assessed by Kubo et al. (34), even following multiple tumor rechallenges, indicating no reduction of antitumor effect. Even though the interaction with EPHRIN B2 should induce weak proliferation*, in vitro* results refuse this idea and do not promote proliferation in RMS cells (55). Another considered point was the possible effect off immunomodulatory effect of the P3F fusion gene, which undergoes some RMS variants, on CAR-T activity. Nevertheless, this translocation product did not modulate the EPHB4-CAR-T activity (56).

After substantiating that EPHRIN B2 could bind EPHB4 mouse receptors, the antitumor effect and off-tumor on-target toxicity were *in vivo* verified with RMS tumor xenograft models. The results showed decreased tumor growth rates and prolonged survival in treated animals with EPHB4-CAR-T compared with anti-CD19 CAR-T control without any sign of adverse effects (55). These promising results have led to the generation of novel studies, and future clinical trials are being proposed.

### FLT3L CAR-T cell

Acute myeloid leukemia (AML) is still a rare malignancy but represents a third of all diagnosed leukemias. Two ligand-based CAR-T cells have been proposed against AML, FLT3L CAR-T and granulocyte-monocyte colony-stimulating factor receptor (GMR) CAR-T (32, 33). Approximately 30% of AML cases have mutated the FMS-like tyrosine kinase-3 (FLT3), mainly internal tandem duplication (ITD) mutations that lead to constitutive activity of tyrosine kinase domain (TKD) and promote, *via* different signaling pathways, the progression of AML with poor prognosis (57, 58). As scFv-based CAR-T targeting FLT3 has no optimal results, Wang et al. (32) developed a second-generation ligand-based CAR-T cell with the FLT3 ligand (FLT3L) as the recognizing domain (32, 59).

FLT3L CAR-T cell can specifically recognize FLT3-positive cells, and *in vitro* studies have proven their cytotoxic efficacy against 10 different primary AML cell lines, five with FLT3-ITD and five with wild-type (WT) FLT3 expression. Moreover, treated mice showed longer survival, but results also revealed that recognition seemed to be independent of FLT3 levels on cells, relying on the FLT3 genotype (32).

Cytotoxicity efficacy was proven *in vitro* against 10 different primary AML cell lines, five with FLT3-ITD and five with WT FLT3 expression. FLT3L CAR-T cell can specifically recognize FLT3-positive cells and display cytotoxicity. *In vivo* experiments verify this idea, since treated mice showed longer survival. In fact, results revealed that recognition seemed to be independent of FLT3 levels in cells but relied on the FLT3 genotype (32).

FLT3L-FLT3 interaction allows dimerization and phosphorylation of FLT3 and activation of downstream signaling pathways that end up in cell growth and survival (60, 61). Since FLT3L CAR-T allows ligand-dependent activation, it can stimulate this phosphorylation and may promote cell growth in FLT3 WT. Thus, FLTL3 WT is less sensitive to CAR-T cytotoxicity. Otherwise, FLT3-ITD is constitutively activated (ligand-independent) developing different phosphorylation profiles that are more sensitive to CAR-T therapy when FLT3 CAR-T interacts. This may allow the CAR-T therapy the ability to distinguish between both types of cells, being more lethal for mutated FLT3. So, distinguishing the receptor by genotype can be a novel strategy with potential benefit in such types of tumors (32).

### GMR CAR-T cell

Another different approach for AML treatment is GMR CAR-T cells. The granulocyte-macrophage colony-stimulating factor (GM-CSF) is an immunomodulatory cytokine capable of tuning the phenotype of myeloid cells but also T cells through myeloid intermediaries (62). Its main target is GM-CSF receptor (GMR), composed of two subunits: α subunit (CD116) that is present in normal and AML and juvenile myelomonocytic leukemia (JMML) myeloid cells and β subunit (CD131) shared with IL-3 and IL-5 receptors. Recent studies revealed that GMR can be found as complexes of two α subunits (low-affinity receptors) or both α and β subunits (high-affinity receptors) (33).

AML expresses both complexes, and since CD116 is overexpressed in more than 60% of AML, mainly in those with poor prognosis, Saito et al. (33) proposed a second-generation ligand-based CAR-T cell targeting GMR (33, 63) after they demonstrated antiproliferative effects of the same construct against JMML (63). Different CAR-Ts were built and evaluated to enhance this effect against AML. Referring to a previous work by López et al. (64), they used GM-CSF as a binding region (33) mutated in residue 21 that plays a key role in the functionality of the cytokine but not affecting the binding (64). After screening analysis of several mutated GM-CSFs, E21K and E21R, both had increased antitumor response. *In vitro* and *in vivo* results revealed E21K mutation as the one with durable *in vitro* cytotoxicity and complete suppression of the progression of CD116+ AML cells *in vivo*, correlating strongly with the CD116 levels in tumor cells. These may appear to conflict with other reported data in which E21K-mutated GM-CSF had a reduced binding capacity to high-affinity receptors but maintained the binding capacity to low-affinity receptors, leading to less AML interaction than scFv-CARs (33, 64). Although the mechanism has not been identified yet, it seems that the reduction of time interaction with receptors would enhance T-cell stimulation (65). Despite all of these important results, off-tumor adverse effects were not tested and this would be necessary to evaluate for further applications (33).

### Thrombopoietin-based CAR-T cell

Thrombopoietin (TPO) is a hematopoietic growth factor produced not only by the liver but also in the bone marrow and kidney niches. TPO is defined as a natural ligand to the myeloproliferative leukemia (MPL) receptor, also known as CD110. Overexpression of MPL has been characterized as a negative prognosis factor for AML progression due to the effects of the associated signaling, such as Janus Kinase 2 (JAK2)/STAT5, Phosphoinositide 3-kinase (PI3K)/Protein kinase B (AKT), and proto-oncogene, serine/threonine kinase (Raf1)/Mitogen-activated protein kinase (MAPK) (36).

The TPO/MPL pathway is essential for the survival and self-renewal of leukemia stem cells (LSCs) and hematopoietic stem cells (HSCs) and is therefore involved in the progression of AML. For this reason, Zoine et al. (36) proposed a second-generation ligand-based CAR-T cell using the biologically active region of the TPO protein to target the MPL receptor. The outcomes showed not only a significantly specific cytotoxicity against MPL+ AML cell lines *in vitro* but also satisfactory results in murine AML xenograft models. Notably, on-target off-tumor toxicities were detected in the bone marrow compartment during the trials. The authors justify that bone marrow toxicity could be advantageous for the model, as most patients with AML receive a bone marrow transplant and treatment with TPO-based CAR-T cells may be helpful to replace the adverse effects of pretransplant conditioning regimens.

### IL-10-based CAR-T cell

Among the diverse ways of drawing an antitumor response in AML is that based on the IL-10 receptor (IL-10R). IL-10R is a receptor composed of four members, two alpha (IL-10RA) and two beta (IL-10RB) molecules, being hematopoietic-specific and ubiquitous, respectively. Published data infer that the IL-10/IL-10R pathway, when with aberrant function, is involved in promoting the stemness of AML cells (37). For this reason, it seems reasonable to validate the CAR-T response against IL-10R. To this end, Chen et al. (37) designed a second-generation ligand-based CAR-T cell using IL-10 as a binding motif and assessed the degranulation and cytokine secretion from T cells and killing of the AML-targeted cells in culture. Following good *in vitro* results, they assessed the product in a murine AML xenograft model and obtained prolonged survival in treated models compared with those that did not undergo CAR-T treatment.

### CD27 CAR-T cell

CD70 is the membrane-bound ligand of the CD27 receptor, which belongs to the TNF receptor superfamily. This interaction is considered a potential target to address CD70-positive malignancies, such as diffuse large B-cell lymphoma and follicular lymphoma, as well as AML, since CD70 is expressed on most of its leukemic blasts, while its expression is low or absent in normal bone marrow samples (66). Importantly, CD70 expression is transient and restricted to a subset of highly activated T, B, and dendritic cells under physiological conditions, playing a role in T-cell activation. However, it is not essential for the development and maintenance of a functional immune system.

In this context, Sauer et al. (38) developed a first-generation CAR-T cell based on a ligand, CD27z-CAR, which uses the full-length CD27 cDNA as a recognition domain. Additionally, their research incorporated the design of several CAR-T sequences with the CD70-specific scFv to compare reactivity against the target.

All CAR-T cell populations mediated cytotoxicity against CD70-positive tumor cell lines but not CD70-negative cells in *in vitro* assays (38). However, because the efficacy of CAR-T cells is determined not only by their cytolytic activity but also by their ability to proliferate after the tumor challenge, they were subjected to successive cocultures. The results showed differences in their ability to kill and proliferate during successive cocultures, with CD27z-CAR able to kill tumor cells during five consecutive cocultures in at least two of the four donors in contrast to other CAR-T cell populations, as well as the highest production of T helper 1 (TH1)-type cytokines, such as Interferon (IFN)-γ and TNF-α (38).

Following these results, the same research evaluated the CD27z-CAR *in vivo* effects in CD70-positive AML murine xenograft models. Their results demonstrated an efficient ability to control leukemic growth, leading to complete leukemia remission in all mice by day 21 (38). Furthermore, they could demonstrate a significant expansion of the transduced T cells in the *in vivo* models, thus corroborating the relationship between the administered therapy and the remission of pathology.

## Ligand-based CAR-T cell clinical trials

Although anti-CD19 scFv-based CAR-T therapies have clinically succeeded, as several products have already been approved (12, 13), the reality is that limited clinical information is still available for other strategies, as could be for ligand-based CAR-T cells. However, a few phase I/II clinical trials are currently trying to elucidate the safety and bioactivity of different approaches.

### IL-13-zetakine CAR-T cell

One of the most hopeful proposals is focused on treating central nervous system (CNS) solid tumors, such as glioblastoma multiforme (GBM) (67–69). GBM is one of the most lethal primary brain tumors, and its outcome remains poor. High-grade glioblastoma does not respond to standard treatments such as surgery or chemotherapy mainly because of tumor heterogeneity (70). Diverse differentiation status has been found in GBM cell populations: stem-like cancer-initiating cells (GSCs), expressing stem cell markers and maintaining certain self-renewal capacity, and differentiated glioblastoma cells (71). It has been proposed that GSCs are responsible for this lack of response because of their natural resistance to conventional treatments (72).

IL-13 receptor α2 (IL13Rα2) is demonstrated to be expressed within 50%–80% of GBM cells, independently of differentiation status, but not significantly expressed in normal CNS tissue. IL13Rα2-positive tumors are associated with a worse prognosis, hypothetically owing to IL-13/IL13Rα2 interaction (73–75). IL-13 is an immunomodulatory cytokine that promotes apoptosis and transforming growth factor alpha (TGF-β) secretion when it interacts with IL13Rα1/IL-4Rα high-affinity heterodimer. Alternatively, IL-13 has a higher affinity to IL13Rα2 but does not induce intracellular signaling (76). Therefore, overexpression of IL13Rα2 in GBM may reduce proapoptotic signaling and promote cell survival (75).

Considering these facts, IL13Rα2 is a suitable candidate for different treatments as is IL-13-zetakine (77, 78). This product is an adoptive T-cell therapy engineered with a CAR structure whose recognition domain is IL-13 cytokine, which contains the E13Y mutation, for targeting IL13Rα2. The importance of this mutation relies on reducing the affinity to IL13Rα1/IL-4Rα heterodimer but increasing IL13Rα2 binding compared with WT IL-13 (77). The aim is to specifically redirect the cytotoxic activity of T cells to GBM cells that overexpress this TAA compared with normal CNS cells.

During the last few years, a first-generation IL-13 CAR-T was developed and tested, obtaining a sustained cytotoxic response to both cancer-initiating cells and differentiated GBM cells *in vitro*. Also, there was evidence of antitumor activity and limitation of the progression of established IL13Rα2-positive tumors in xenograft mice without clear collateral damage on healthy tissue (77, 78). With these results, Brown et al. (79) conducted a first-in-human pilot clinical trial (NCT00730613) to assess the activity and safety of IL-13-zetakine after intracranial delivery in three patients with recurrent GBM. Indeed, two out of three showed transient antitumor activity in the absence of severe adverse events. Although the survival rate was 11 months, the small cohort denied the capacity to establish the therapy survival benefit (67).

Aware that the CAR-T response needs to be improved, Brown et al. (80) started to tune the IL-13-zetakine structure. Thus, the second-generation CAR-T cell was developed using 4-1BB as the costimulatory domain and CD3ζ as the intracellular signaling domain (80). Preclinical results showed an enhanced response. As the first-generation IL-13 CAR-T activity was transient, and its persistence was limited, one of the aims was to analyze the antitumor response and cell persistence of the second-generation one. For this reason, a current phase I clinical trial (NCT02208362) (81) studies the activity, adverse effects, and best dose of these CAR-T cells. In fact, one patient has reported a transient complete response after complete CAR-T dose administration with important improvements in the quality of life for up to 7.5 months (68). The trial is still ongoing, but this case appears to be a great hope. Another clinical trial has just started (NCT04661384) to test this CAR-T therapy in patients with leptomeningeal disease from glioblastoma, ependymoma, or medulloblastoma, but the results are not expected to be analyzed until December 2022 (69).

### Pan-ErbB CAR-T cell

The ErbB receptor family comprises a synergistic dynamic signaling network composed of four members, EGFR/ErbB-1, ErbB-2/NEU/HER2, ErbB-3/HER3, and ErbB-4/HER4 (49). After ligand-dependent stimulation, diverse homodimer or heterodimer combinations may occur (50). ErbB-2 is demonstrated to be the preferred member for dimer formation, while ErbB-3 pairing is essential, because of its lack of intrinsic tyrosine kinase (TK) activity. Upon dimer activation, tissue development, proliferation, and differentiation are promoted (50).

Several studies have revealed aberrant expression or function of some ErbB receptors, mainly ErbB-2 dimers with ErbB-1 or ErbB-3, as a determinant of the pathogenesis of many malignancies, such as mesothelioma, epithelial ovarian carcinoma (EOC), or head and neck carcinoma [head and neck squamous cell (HNSCC)] (82–85). Consequently, there is a considerable interest in targeting ErbB family members, but problems of selective pressure and tumor resistance have been emerging due to the overexpression of non-targeted receptors (82).

To circumvent this, diverse approaches are currently trying to redirect their mechanism toward two or more ErbB dimers to prevent the signaling network from escaping and continuing tumor progression. This idea includes T1E28z or pan-ErbB CAR-T cell, a second-generation CAR-T therapy that includes T1E as a binding moiety for treating many epithelial malignancies (83).

T1E is a chimeric polypeptide that takes benefit from different ErbB ligand properties: epidermal growth factor (EGF) and TGF-α selectively bind to ErbB-1 with high affinity but weaker or no affinity for ErbB-2/ErbB-3 heterodimers, respectively. Structural analysis of both revealed that EGF is unable to bind ErbB-2/ErbB-3 with high affinity because of the lack of essential amino acids in the N-terminal region, whereas TGF-α cannot bind despite having these crucial residues (86). Thus, a chimera was developed introducing N-terminal linear region of TGF-α into the EGF C-terminal sequence, resulting in high affinity for ErbB-2/ErbB-3 maintaining ErbB-1 specificity (87). ErbB-4 heterodimer binding was also reported. This made T1E a promiscuous ligand ideal for multitargeting ErbB dimers, preventing antigen loss and signaling compensation.

Davies et al. (83) engineered T cells with T1E28z and evaluated its binding capacity, resulting in eight of nine possible ErbB homo and heterodimers, with most affinity detected against cells that coexpressed ErbB-1 and ErbB-2 (83). One of the main challenges of CAR-T therapies is the enrichment and expansion of T cells. For this reason, they also introduced a chimeric cytokine receptor named 4αβ, in which IL-4 receptor-α ectodomain has been coupled to the shared β chain used by IL-2/15 (88). With IL-4, T cells receive a potent and selective stimulation, allowing better expansion. Preclinical studies have revealed that T4 immunotherapy (CAR-T combining T1E28ζ and 4αβ chimeric receptors) achieves a relevant antitumor response in HNSCC, EOC, and malignant mesothelioma *in vitro* (83–85).

Considering that T1E polypeptide can efficiently bind to ErbB mouse receptors, *in vivo* efficiency and toxicity were tested in diverse immunocompromised xenograft mice, including all three malignancies mentioned above (89). T cells elicit antitumor activity in the absence of relevant toxicity when delivered intratumorally or intraperitoneally at a moderate dose. Nevertheless, after high-dose intraperitoneal delivery, cytokine release syndrome (CRS) appeared, providing evidence that intratumor administration seems to be the safest route for solid tumors and that CRS dose-dependently appeared (83, 89).

To build on this, van Schalkwyk et al. (90) designed a phase I clinical trial (NCT01818323) to assess the safety of T4 immunotherapy to treat HNSCC that is not suitable for conventional active therapy. Primary results are expected to be published in April 2022. If results are robust, other clinical approaches should be initiated to evaluate CD4+ CAR-T therapy against other malignancies, such as EOC or malignant mesothelioma (91).

### AUTO2: APRIL-based CAR-T cell

MM represents 13% of all hematologic cancers, and it is characterized by extreme growth of malignant plasma cells (PCs) in the bone marrow, aberrant production of monoclonal immunoglobulin, and immunosuppression, among others (92, 93). Over the past decade, autologous stem cell transplants, proteasome inhibitors (PIs), and immunomodulatory drugs (IMiDs) have significantly raised survival rates, and moAbs further improved relapsed/refractory multiple myeloma (RRMM) outcomes (93–95). Nonetheless, overall survival is extremely reduced in patients with RRMM after IMiDs or IP treatments. Therefore, there remains a need for new approaches that could lead to durable remissions in MM patients, especially in RRMM (94).

Although the CARTITUDE-1 trial seems to have recently promising results (18), treatment of MM still involves many challenges, focusing on TAA detection. Since CD19 has a reduced expression in malignant cells, some RRMMs appear to be CD19-negative and other well-defined antigens (CD56 or CD38) have expression levels in other tissues, then other antigens need to be validated (95).

BCMA is another suitable candidate for CAR-T therapies because it is absent on hematopoietic stem cells but selectively expressed on PCs, and BCMA is almost present in MM cells. After the first anti-BCMA CAR-T cell trial, remission was reported in four of 12 patients, but high doses were required for persistent remission due to the low expression of BCMA in MM cells. Moreover, similar to CD19 therapies, tumor escape by downregulation of targeted BCMA was reported (96, 97).

Considering this, Lee et al. (98) attempted overcoming low target density and antigen escape targeting two TNF-receptor superfamily members, BCMA and a transmembrane activator and calcium-modulator and cyclophilin ligand interactor (TACI) (98). Both are coexpressed on the majority of PC and MM cells and may play a similar role in providing PC with survival signaling (35). For this reason, a bispecific third-generation CAR was constructed using a murine truncated version of a proliferation-inducing ligand (APRIL), a natural ligand for both BCMA and TACI with nanomolar affinity (99, 100). The final product, AUTO-2, is retrovirally transduced to produce CAR-T cells expressing APRIL-CAR-T and the RQR8 switch system that acts as a marker but also as a suicide molecule when adverse effects of the therapy occur (101, 102).

The preclinical evaluation demonstrated cytolysis at low levels of target antigen, even when the BCMA expression was downregulated or lost. These data were confirmed *in vivo*, where an improved disease control compared with scFv-based CAR was observed (101), but the problem still was the need for considerable T-cell doses to achieve relevant responses with a short follow-up duration. A phase I/II clinical trial (NCT03287804) (102) was initiated to test the safety and efficacy of AUTO2 in RRMM patients. Phase I showed that eight of 11 (81.9%) treated individuals achieved expansion and persistence of CAR-T cells in peripheral blood, while four of 11 (36.4%) had a complete/very good/partial response up to 2 years. However, the average death during the trial was eight of 11 (72, 73), so the duration of response could not be quantified, and thus, phase II was not initiated.

Even though the results of the clinical trial seemed to show a glimmer of hope, APRIL-based CAR-T cells required better optimization. After evaluation of all of these studies, Schmidts et al. (35) generated a second-generation CAR where they changed the extracellular domain of AUTO2 for a tri-APRIL binding moiety (TriPRIL) (35). The study hypothesized that preserving the trimeric form of the natural ligand would increase the binding affinity and efficacy against MM cells. Also, they used human APRIL so it would reduce immunogenicity. Indeed, data revealed that antitumor activity was enhanced *in vitro* against BCMA+ and BCMA- cells and *in vivo* with xenograft models compared with monomeric APRIL-based CAR-T cells (35). Therefore, TriPRIL CAR-T therapy holds promise for treating MM, including the absence of BCMA. Further clinical trials will be required to elucidate its potential clinical benefits.

### Beyond ligand-based CAR-T

**Figure 3:**
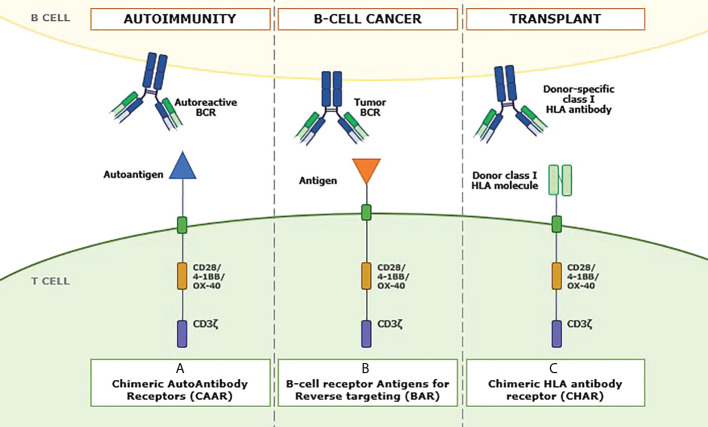
Role of ligands in other antibody-mediated diseases. Recently, new approaches have emerged in which different ligands linked to antibody-like structures can be used to address specific pathologies. The sequence of these products shares similar domains to CAR-Ts but changes the extracellular domain. In panel **(A)**, a chimeric autoantibody receptor (CAAR) can be seen in which an autoantigen is incorporated as a recognition domain of the CAAR to redirect it toward autoantibodies on the autoreactive B-cell surface, thus facing autoimmune responses. Panel **(B)** shows the structure of a B-cell receptor antigen for reverse targeting (BAR). The recognition domain includes an antigen specific to the single B-cell receptor (BCR) clonally specific of each B-cell tumor. Finally, panel **(C)** represents an example of chimeric HLA antibody receptor (CHAR), where molecules of the class I HLA system replace the conventional recognition domain to direct the transduced T cell toward anti-HLA antibody-producing B cells to cope with posttransplant immune rejection.

Although this review focuses mainly on what the literature refers to as ligand-based CAR-T cells, it is necessary to mention other approaches in which ligands are incorporated as the binding moiety of the CAR structure. In this regard, we found the so-called chimeric autoantibody receptor (CAAR) (103), B-cell receptor antigen for reverse targeting (BAR) (104), and chimeric HLA antibody receptor (CHAR) **(** **Figure 3** **)**.

Treatments for autoimmune diseases specifically eliminate self-reactive cells while preserving protective immunity (103). However, this premise is proving difficult to implement, as both autoimmunity and cancer are closely related, requiring optimal management of autoimmune therapies to prevent cancer development due to general immunosuppression (105). For this reason, novel approaches try to avoid the classic issues associated with autoimmunity treatments.

In this sense, CAAR molecules consist of a chimeric immunoreceptor that includes an autoantigen as the extracellular domain. This technology directs the modified T-cell response toward autoreactive memory B cells expressing autoantibodies as their surface immunoglobulins (sIg-BCR), or autoantibody-producing PCs, which are autoantibody-secreting cells (103). This would generate selective therapy against reactive immunity, thus avoiding a general suppression of the abovementioned protective immunity. This strategy has been used by Ellebrecht et al. (103), who have constructed a CAAR T cell using the different forms of the Dsg3 autoantigen target of pathological autoantibodies present in a significant percentage of patients with pemphigus vulgaris (PV). Their results expect potential benefits that these CAARs could bring to the treatment of autoimmune diseases.

BCR signaling has been identified as an important pathway in B-cell lymphomagenesis, and there is increasing evidence that antigenic stimulation of the BCR is a trigger for proliferation. Several autoantigens, such as ARS2 and LRPAP1, have been proposed as stimulatory ligands of the BCR and its pathway in one quarter of diffuse large B-cell lymphomas (DLBCLs) and almost half of the mantle cell lymphomas (MCLs), respectively (104–106).

From the study of these BCR antigens arise structures defined as BAR. BAR-bodies were initially designed with the idea of conjugating toxins to these BCR antigens. One example is the research led by Thurner et al. (107), where it is shown that LRPAP1-based BARs conjugated to *Pseudomonas aeruginosa* exotoxin A toxin are internalized and specifically kill MCL cells with LRPAP1-reactive BCRs by inducing apoptosis.

Further research has led to the construction of an antibody-like structure that incorporates the sequence of these identified BCR antigens, or at least their BCR-binding epitope, replacing the variable fragments of the scFv heavy and light chains, with the aim of transducing T cells and targeting malignant B-clones with unique specificity for these BCRs responsible for tumor expansion (104, 106). Since approaches such as that of Bewarder et al. (106) that uses the BCR antigen to target MCL cells have exclusive specificity for cells with the specific surface BCR, they do not only represent a strictly tumor-specific approach but can also be expected to be more effective but less toxic than the currently available CAR-T cells with specificity for CD19, as they should not affect other cells and should work equally well in the presence of autoantibodies against the antigen in question.

In the same line as CAARs and BARs, T cells that express CHAR with the ability to kill B cells that produce donor-specific class I HLA antibodies are being developed to treat antibody-mediated rejection in the field of solid organ transplantation. One of the main problems in solid organ transplantation is the presence, or *de novo* generation, of donor-specific antibody anti-HLA molecules (anti-HLA-DSA), which is associated with a high risk of antibody-mediated rejection (108). Our thinking has changed from considering rejection as a primarily T cell-mediated process. Insufficient control of the humoral arm of a recipient’s immune system by current immunosuppressive regimens is now the pathogenic factor primarily responsible for allograft dysfunction and loss (109). This new CHAR could be a therapeutic approach for personalized desensitization of HLA-sensitized recipients and even for antibody-mediated rejection in solid organ transplantation.

## Discussion

CAR-T cell-based therapeutic strategies allow the production of significant numbers of tumor-specific reactive T cells, resulting in potent responses that can lead to the elimination of tumor cells expressing the target antigen (1). As mentioned above, those approaches are being considered as one of the further progress in the field of antitumor therapies, even including several commercial products available for clinical use (12–16). Specifically, it is the conventional CAR, with the scFv fragment as the recognition domain, that is mostly proposed, since they were the first to appear and to get results. In any case, scFv-based CAR is not exempt from limitations and concerns, such as immunogenicity or toxicity (5, 24). For this reason, ligand-based CAR-T cells are now emerging as a suitable alternative to address them (110). They are presented as an alternative therapy because most of the preliminary and clinical studies conducted to date show similar results in terms of activity and efficacy (110).

It is relevant to highlight the commonalities between the two technologies. The proof of concept involves engineering of autologous T cells, allowing the CAR expression on the cell surface, so that the modified cells can acquire tumor specificity. Since the same intracellular signaling domains are used, upon recognition of the target molecule, the internal signaling necessary to enable T-cell activation and expansion will be triggered (22, 23).

Both approaches share HLA-independent target recognition, which allows for less restriction in the recognition of what they are meant to act against. The main difference lies in the interaction on which target recognition depends. While scFv-based CAR-T cells rely on moAb–target binding, which is defined as a higher-affinity interaction, ligand-based CAR-Ts use ligand–receptor binding, which is presumably a lower-affinity interaction (28). Using the properties of this modification in the CAR structure, the aim is to mitigate the issues detected after administration of conventional CAR-T therapies.

### Immunogenicity

CAR-T cells have the potential to trigger both cellular and humoral immune responses against non-self-components of the CAR structure, but its clinical implication remains poorly investigated and exhibits great variability depending on the CAR-T and type of tumor (24).

Human anti-mouse antibodies (HAMAs) against mouse-derived scFvs have been detected in a subset of patients. Anti-idiotype antibodies are also reported, directed toward specific regions of scFv binding sequence, since the hypervariable region (the idiotype and the allotype determinants) of the scFv are highly immunogenic. Additionally, antibodies against CAR peptides originated from the fusion of the domains that make up its structure can also be found (24, 25). Regarding the cellular immune response, specific cytotoxic T cells could arise from the processing and cross-presentation of foreign peptides of the CAR structure. Finally, immune response could also be triggered by residual elements from gene transfer viral vectors, which are inevitably immunogenic (24).

Despite the lack of conclusive evidence, it is inferred that these elements may interfere negatively with CAR-T cell activity by either neutralizing their recognition capacity, causing loss of CAR membrane expression, or directly increasing CAR-T cell apoptosis (24, 25). Immunogenicity arising from the non-human origin of scFv could be reversed using human moAbs, but the full ability and a sufficient human moAb library to obtain them are not currently available. Also, humanized scFv can be an option, but problems exist with cell surface stability, dimerization, and aggregation that limit the desired cytotoxic activity (26, 110).

Ligand-based CAR-T cells have been proposed to dodge these adverse effects considering that natural human ligands are used to replace the scFv region, as non-human sequences are eliminated, and the immunogenicity of the product is reduced (both HAMA and cytotoxic T cells). It is worth mentioning that the reduction in immunogenicity will be greater the more original ligand sequence is included (full-length sequence) in the final recombinant molecule (26, 31, 35). Antibodies directed against the region of the fused domains and immunogenicity of viral vector peptides will still exist, but ligand-based CAR-T cells may be less likely to prompt immune responses (35).

Despite that fact, it requires further investigation to elucidate the possible benefit of using ligand-based CAR-T cells to reduce immunogenicity, as there is a lack of results to prove it.

### Tonic CAR signaling and scFv instability

Throughout the design process of scFv molecules, many studies have highlighted problems of oligomerization either as part of a CAR structure or in a soluble form. Oligomerization occurs mainly through a process of domain swapping, where the V_H_ region of one scFv is incorrectly associated with the V_L_ region of another scFv (26, 111). This causes the aggregation of CAR structures, which will result in dysfunctionalities leading to tonic signaling through constitutive activation *via* the signaling described above. Not to mention that these oligomerizations could lead to problems in target recognition by the CAR (111).

CAR-transduced T-cell tonic signaling is widely described in many investigations. In addition to aggregations of scFv, high levels of cell surface CAR expression, the addition of endogenous TCR-associated signaling, and the incorporation of certain intracellular signaling domains into the CAR sequence can trigger constitutive cytokine release, prolonged and excessive expansion, and thus further T-cell exhaustion (26, 111). However, natural ligands are probably more stable and have a lower risk of dimerization and domain swapping if no multimeric ligands appear, and these will require further study. Thus, ligand-based approaches would reduce potential tonic signaling that prevents early exhaustion of CAR-T therapy, thus prolonging its functionality and thus improving the probability of success of the intervention when administered in *in vivo* models.

### Affinity-tuning and toxicity management

Another potential advantage of ligand-based CAR-T cells is their capacity to multitarget (110). The native forms of ligands that are proposed for CAR structure often can bind to different receptors, being bispecific or trispecific. Recent publications consider that one of the main drawbacks encountered is the relapsed/refractory state of some malignancies after CAR-T infusion mainly due to downregulation of the specific antigen against which the cytotoxic activity is directed (antigen loss) (21, 31). Given that scFv has a single specificity for a particular peptide, ligand-based CAR-T cells may provide a safeguard against antigen loss, anticipating one of the possible mechanisms of tumor evasion (112). For instance, the APRIL-CAR proposed by Lee et al. (98) has bispecificity for BCMA and TACI, leading to increased recognition and lysis of MM cells, either BCMA+ or BCMA-, compared to an scFv-based CAR-T cell directed against BCMA that does not prevent the proliferation of BCMA- tumor cells (35, 98).

This potential benefit from ligand-based CAR-T cells must be balanced against the toxicity problems that have already been described in some patients when treated with scFv-based CAR-T cells (5). Specifically, these off-tumor on-target toxicities arise because target molecules can be constitutively expressed in healthy tissues, causing harmful activity by altering normal tissue functionality (3). If a product with bispecificity or trispecificity is infused, the potential number of off-target sites at which it can act increases significantly, thereby increasing the risk of adverse effects (4, 21, 35). Therefore, this promiscuous binding could be also analyzed as a disadvantage of the ligand-based design if other known or unknown binding partners exist in healthy tissues, leading to off-tumor on-target toxicities. For this reason, these potential toxic effects of ligand-based CAR-T cells need to be carefully explored in animal models.

However, this could be countered by considering the sensitivity of the CAR interaction toward the recognized molecule. Normally, the process of production of scFv regions depends on somatic hypermutation mechanisms, in which molecules with the highest affinity for the target are selected (113). Thus, at low concentrations of the target molecule in the tumor, the CAR-T cell is able to reach the activation threshold and trigger the cytotoxic response (31, 114). However, this characteristic of high-affinity CARs makes them poorly able to discriminate between target cells with various levels of antigen expression. Considering that tumors tend to overexpress certain molecules above basal levels present in other tissues (82), this means that the CAR-T cells activate the cytotoxic response not only toward the tumor but also toward other healthy tissues, increasing the risk of off-tumor on-target toxicities. Finally, scFv-CAR-T cells have constitutive basal activation of CAR signaling by their extracellular domain, increasing the off-target effects of the therapies and the earlier exhaustion of the T cell (31).

By using ligands and not antibody chains as the target-binding molecule, a lower-affinity interaction will be achieved (110). Additionally, the ability to modify native ligand sequences also offers some flexibility in CAR binding. An example is the GM-CSF-CAR, where mutations are introduced into the target-binding domain so that the product has less affinity toward its target (33). By reducing the affinity, a high TAA expression level will be needed to activate the T cell, increasing the selectivity of ligand-based CAR-T therapy against tumor cells. However, previous studies have shown that the affinity of a CAR toward its target is inversely proportional to the activation threshold of the T cell, although the mechanism is not yet fully described (114). Thus, by modifying the ligands, we can generate CARs that are less affine but induce greater cytotoxic activity on the tumor.

Ultimately, if the potential benefits associated with the ability to prevent and/or reduce tumor evasion of the immune response can be balanced during *in vitro* development and testing in *in vivo* models with a thorough analysis of CAR-T interactions with known and undescribed targets, if, in addition, possible modifications of the ligand used to modulate the cytotoxicity of the CAR-T product can be described, and if immunogenicity issues are resolved, we may be talking about a therapeutic alternative that will potentially discriminate tumor from healthy tissue, be tumor-specific, and reduce the risk of adverse effects (31, 110).

### Challenges ahead for ligand-based CAR-T cells

Although the use of natural ligand-based CARs presumably has many advantages, these alternative CAR designs have their own limitations. These include the potential for off-tumor toxicity, unwanted target-associated signaling, and possible interference with the physiological interaction between the endogenous ligand and the target.

As has already been mentioned in this review, the possibility of ligand binding to different targets may lead, on the one hand, to a reduction in the ability to evade the antitumor response, at the same time, it may trigger on-target off-tumor toxicities by increasing the range of possible interactions outside the tumor (4, 21). Although this can be contrasted with the modulation of the interaction affinity, making the therapies more selective, this aspect must be widely considered in the testing phases in animal models. It is worth mentioning that toxic effects are also present in conventional CAR-T therapies, and therefore, it is a pending task for all CAR-T cell therapies.

Another aspect to consider is the possible unwanted signaling that may be generated in the target cell because of the interaction with the ligand fused to the CAR. For example, if the ligand plays a role in cell proliferation and survival, their interaction could increase tumor growth. This is a very preliminary approach that requires further study, but a possible alternative would be to introduce modifications in the ligands that prevent signal transduction in the target, as proposed by Saito et al. (33), who introduced mutations in GM-CSF at residue 21, a key to the functionality of the ligand but maintaining the binding capacity.

Finally, possible interference of endogenous ligands on the interaction of the ligand that is used as the CAR binding moiety should also be considered, generating a competition between both for binding to the target (35). This could result in reduced functionality of the CAR-T cell. Although this is presumed to be a mild effect, it should be considered individually as a reason for the study depending on the type of ligand.

## Conclusion

As described in this review, ligand-based CAR-T cells offer several advantages over CARs containing an scFv domain as a binding moiety. Although they should generate greater toxicity problems due to their ability to recognize multiple off-tumor targets, clear advantages are described: 1) they are less likely to provoke an immune response, as the ligands are derived from natural human sequences and therefore fewer murine regions will be present in the CAR structure; 2) somatic hypermutation phenomena are not necessary, thus reducing anti-idiotype antibodies; 3) ligand-based CARs are often able to bind to multiple targets, thus reducing the potential for tumor escape; 4) the nature of the ligands and their binding to the receptor allow for a certain tuning capacity that reduces their sensitivity and enables therapies with a greater ability to discriminate between tumor tissues, which tend to overexpress the target, and healthy tissues; and 5) less tonic signaling and longer lasting functionality should be detected associated with a reduction in the probability of ligand aggregation.

Ideally, ligand-based CAR-T therapies appear to be proposals that would improve the safety profile of CAR-T cells and increase cell persistence, maintaining similar levels of response to those achieved with scFv-based CAR-T cells in hematological malignancies and translate these to solid tumors. They should therefore be presented as a significant advance in cancer immunotherapy. Nevertheless, there is still a lack of data and much research to be done to truly elucidate their potential benefit and corroborate their safety profile. Therefore, public and private institutions should invest in the development and testing of these products and technologies. However, there is a conflict of interest, as native ligand sequences cannot be patented like scFv; at the basic research level, it would facilitate the production of therapies, but, for the time being, there is a lack of investment to bring them to clinical trials and to be able to analyze the issues mentioned in this review. It is also particularly important for the scientific community to engage with these types of therapies, as more information is still needed. In conclusion, what emerges from this work is that ligands are intended to offer a future alternative for developing new therapies, but more support and effort will be needed to get results.

## Data availability statement

The original contributions presented in the study are included in the article/Supplementary Material. Further inquiries can be directed to the corresponding author.

## Author contributions

MJ define the main aspects of the review. AR-C, AG, and MM contributed to the conception and design of the review. AR-C organized the database and wrote the first draft of the manuscript. SB-M and AB-I wrote sections of the manuscript. All authors contributed to manuscript revision, read, and approved the submitted version.

## Funding

Thanks to Immunology department (Hospital Clínic de Barcelona) and Institute of Biotechnology and Biomedicine (IBB-UAB) for their help during the writing of this article. This work has been partially supported by grants from the Instituto de Salud Carlos III, Spanish Ministry of Health (FIS PI18/00775, PICI18/00012 and complementary grant for CONCORD-023), Fondo Europeo de Desarrollo Regional (FEDER) “una manera de hacer Europa”, “La Caixa” Foundation (CP042702/LCF/PR/GN18/50310007), and Secretaria d’Universitats i Recerca del Departament d’Empresa i Coneixement, Generalitat de Catalunya, project 2020PANDE00079.

## Conflict of interest

The authors declare that the research was conducted in the absence of any commercial or financial relationships that could be construed as a potential conflict of interest.

## Publisher’s note

All claims expressed in this article are solely those of the authors and do not necessarily represent those of their affiliated organizations, or those of the publisher, the editors and the reviewers. Any product that may be evaluated in this article, or claim that may be made by its manufacturer, is not guaranteed or endorsed by the publisher.

## Glossary

**Table:**

CAR	chimeric antigen receptor
TA	tumor antigen
TAA	tumor-associated antigen
BALL	B-cell acute lymphoblastic leukemia
FDA	Food and Drug Administration
EMA	European Medicines Agency
BCMA	B-cell maturation antigen
AEMPS	Agencia Española del Medicamento y Productos Sanitarios (Spanish drug agency)
scFv	single-chain variable fragment
moAb	monoclonal antibody
OS	osteosarcoma
EGFR	epidermal growth factor receptor
FLT3L	FMS-like tyrosine kinase 3 ligand
FLT3	FMS-like tyrosine kinase 3
AML	acute myeloid leukemia
GM-CSF	granulocyte-monocyte colony-stimulating factor
GMR	granulocyte-monocyte colony-stimulating factor receptor
JMML	juvenile myelomonocytic leukemia
TPO	thrombopoietin
MPLR	myeloproliferative leukemia receptor
EPHB4	ephrin type-B receptor 4
RMS	rhabdomyosarcoma
APRIL	a proliferation-inducing ligand
TACI	transmembrane activator and calcium-modulator and cyclophilin ligand interactor
MM	multiple myeloma
HNSCC	head and neck squamous cell carcinoma
10Fn3	10th type III domain of human fibronectin
TNBC	triple-negative breast cancer
ITD	internal tandem duplication
TKD	tyrosine kinase domain
WT	wild type
CNS	central nervous system
GBM	glioblastoma multiforme
GSC	stem-like cancer-initiating cell
IL13Ra2	interleukin-13 receptor a2
TK	tyrosine kinase
EOC	epithelial ovarian cancer
EGF	epidermal growth factor
TGF-α	transforming growth factor α
CRS	cytokine release syndrome
PC	plasma cell
PI	proteasome inhibitor
IMiD	immunomodulatory drug
RRMM	relapsed/refractory multiple myeloma
TNF	tumor necrosis factor
CAAR	chimeric autoantibody receptor
BAR	B-cell receptor antigen for reverse targeting
sIg	surface immunoglobulin
BCR	B-cell receptor
PV	pemphigus vulgaris
DLBCL	diffuse large B-cell lymphoma
MCL	mantle cell lymphoma
CHAR	chimeric HLA antibody receptor
DSA	donor-specific antibody
HAMA	human anti-mouse antibody
VH	variable heavy chain
VL	variable light chain
