# Corrigendum: Ligand-based CAR T-cell: Different strategies to drive T-cells in future new treatments

**DOI:** 10.3389/fimmu.2022.1078003

**Published:** 2022-11-21

**Authors:** Alejandro Ramírez-Chacón, Sergi Betriu-Méndez, Ariadna Bartoló-Ibars, Azucena González, Mercè Martí, Manel Juan

**Affiliations:** ^1^ Immunology Unit, Department of Cellular Biology, Physiology and Immunology, Universitat Autònoma de Barcelona (UAB), Cerdanyola del Vallès, Spain; ^2^ Laboratory of Cellular Immunology, Institute of Biotechnology and Biomedicine (IBB), Cerdanyola del Vallès, Spain; ^3^ Immunology Department, Hospital Cl´ınic de Barcelona, Centre de Diagnòstic Biomèdic (CDB), Barcelona, Spain; ^4^ Immunology Department, Institut d’Investigacions Biomèdiques August Pi i Sunyer (IDIBAPS) –Fundació Clínic per a la Recerca Biomèdica (FCRB) Universitat de Barcelona (UB), Barcelona, Spain; ^5^ Immunology Department, Hospital Sant Joan de Déu, Barcelona, Spain

**Keywords:** T cells, chimeric antigen receptor (CAR), ligands, receptor, antigen, CAAR, BAR

In the published article, there was an error in the Funding statement. Missing grant supporting this manuscript. The correct Funding statement appears below.

**Funding**

“Thanks to Immunology department (Hospital Clínic de Barcelona) and Institute of Biotechnology and Biomedicine (IBB-UAB) for their help during the writing of this article. This work has been partially supported by grants from the Instituto de Salud Carlos III, Spanish Ministry of Health (FIS PI18/00775, PICI18/00012 and complementary grant for CONCORD-023), Fondo Europeo de Desarrollo Regional (FEDER) “una manera de hacer Europa”, “La Caixa” Foundation (CP042702/LCF/PR/GN18/50310007), and Secretaria d’Universitats i Recerca del Departament d’Empresa i Coneixement, Generalitat de Catalunya, project 2020PANDE00079.”

The authors apologize for this error and state that this does not change the scientific conclusions of the article in any way. The original article has been updated.

## Publisher’s note

All claims expressed in this article are solely those of the authors and do not necessarily represent those of their affiliated organizations, or those of the publisher, the editors and the reviewers. Any product that may be evaluated in this article, or claim that may be made by its manufacturer, is not guaranteed or endorsed by the publisher.

